# P-1597. Timing and Microbiology of Late Positive Blood Cultures in Hematology/Oncology Patients with Persistent Febrile Neutropenia

**DOI:** 10.1093/ofid/ofae631.1764

**Published:** 2025-01-29

**Authors:** Emily A Rosen, Elizabeth M Krantz, Allison Thibodeau, Frank Tverdek, Zahra Kassamali-Escobar, Catherine Liu

**Affiliations:** Fred Hutchinson Cancer Center / University of Washington, Seattle, Washington; Fred Hutch Cancer Center, Seattle, Washington; Fred Hutch Cancer Center, Seattle, Washington; Fred Hutchinson Cancer Center, Seattle, Washington; University of Washington Center for Stewardship in Medicine / Fred Hutchinson Cancer Center, Seattle, Washington; Fred Hutchinson Cancer Center, Seattle, Washington

## Abstract

**Background:**

The optimal frequency of repeating blood cultures in persistent febrile neutropenia (FN) is unknown. We aim to characterize timing and microbiology of positive blood cultures in persistent FN to inform diagnostic stewardship of blood cultures at our cancer center.

**Figure:**
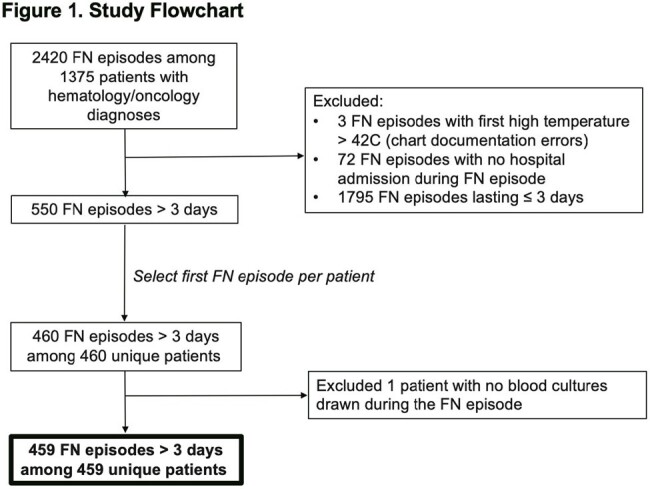

Flow diagram outlining methods for selecting patients to include in the study. Among qualifying FN episodes, only the first FN episode per patient was included in the analysis. Abbreviation: FN (febrile neutropenia).

**Methods:**

This is a retrospective cohort study of adult hematology/oncology patients with a FN episode lasting > 3 days between March 31, 2021 - August 31, 2023. A day of FN was defined as a day with a patient temperature of ≥ 38C and an absolute neutrophil count ≤ 500 cells/µL within 24 hours of the qualifying temperature. Day 1 of FN was the first day a patient met FN criteria; the end of the FN episode was the last day of FN that was followed by ≥ 2 consecutive days where the patient no longer met FN criteria. All blood cultures collected during each FN episode were extracted from the medical record.

**Figure:**
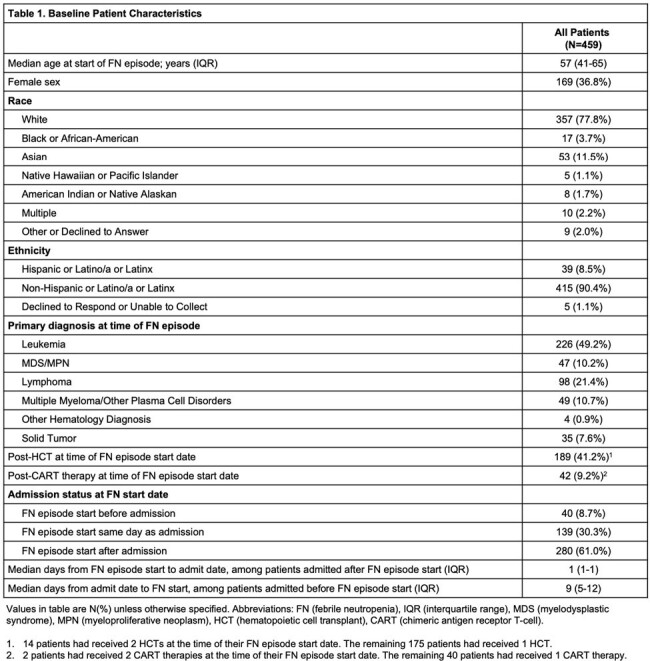

**Results:**

Among 459 patients included (Figure 1), 420 (91%) had a hematologic malignancy (Table 1). The median FN duration was 5 days (interquartile range (IQR) 4-7). A median of 12 blood culture bottles were collected per patient during FN (IQR 8-18). At least 1 positive blood culture at any time during the FN episode occurred in 126 (27%) patients; 23 (5%) patients had ≥ 1 positive blood culture after FN day 3. For 12 of these 23 patients the positive blood culture(s) that occurred after FN day 3 were the first positive cultures during the FN episode. The percent of patients with ≥ 1 positive blood culture declined from 25% on FN day 1 to < 10% on FN days 2-4 and < 5% on FN days 5-8 (Figure 2). Among the 23 patients with positive blood cultures after FN day 3, gram positive bacteria were observed most frequently after FN day 3 (12 patients), with coagulase negative *Staphylococci* occurring most often. Gram negative bacteria and yeast were isolated in blood cultures after FN day 3 from 8 and 5 patients, respectively (Table 2).

**Figure:**
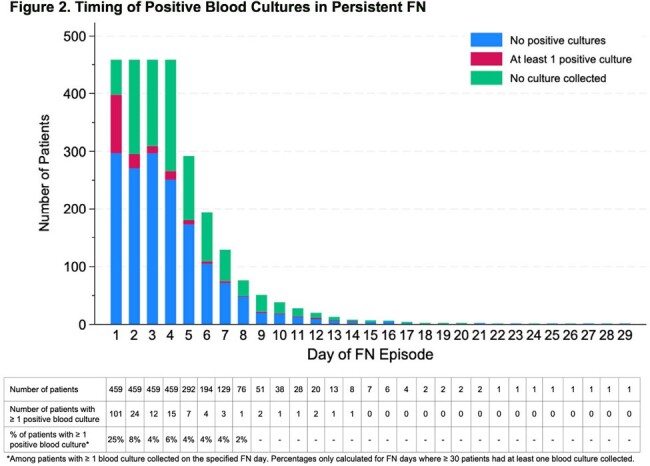

Bar chart depicting the number of patients on each day of FN who had at least one positive blood culture, no positive blood cultures, and no blood cultures collected. The table below the bar chart shows the total number of patients who met criteria for FN on each FN day, the number of those patients with at least one positive blood culture on a given FN day, and the percent of patients on each FN day who had at least one positive blood culture (among the patients who had at least one blood culture collected on the given FN day). Percentages were only calculated for days where there were ≥ 30 patients who had at least one blood culture collected (FN days 1-8). Abbreviation: FN (febrile neutropenia).

**Conclusion:**

Few patients had a positive blood culture beyond day 3 of FN. Gram positive organisms, particularly coagulase negative *Staphylococci*, were most often isolated, raising the possibility that some of these late positive cultures may be skin contaminants rather than true bloodstream infections (BSI). Additional studies are needed to determine optimal frequency of blood culturing in persistent FN and to identify patients at high risk of late onset BSI.

**Figure:**
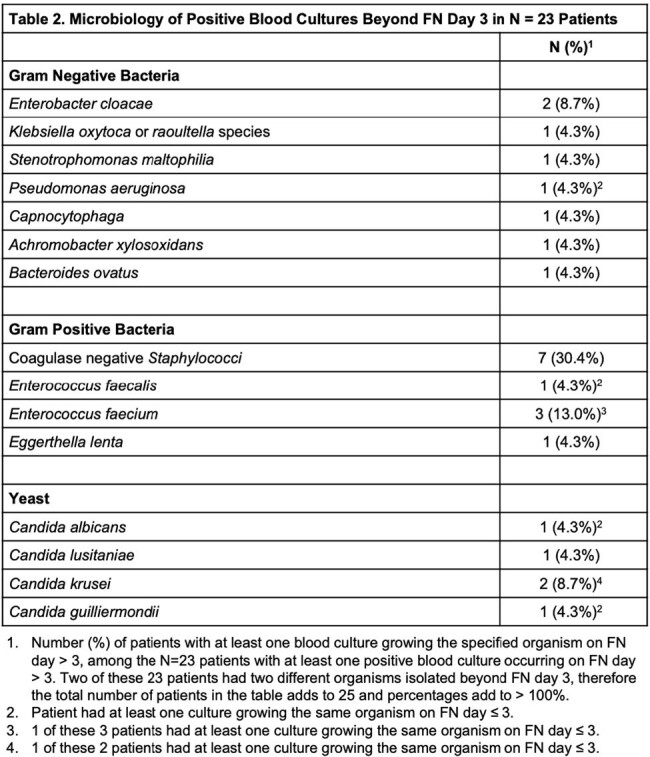

**Disclosures:**

**Frank Tverdek, PharmD**, Merck: Advisor/Consultant **Catherine Liu, MD**, Pfizer: Grant/Research Support

